# Birth weight following pregnancy wildfire smoke exposure in more than 1.5 million newborns in Brazil: A nationwide case-control study

**DOI:** 10.1016/j.lana.2022.100229

**Published:** 2022-03-15

**Authors:** Weeberb J. Requia, Heresh Amini, Matthew D. Adams, Joel D. Schwartz

**Affiliations:** a School of Public Policy and Government, Fundação Getúlio Vargas, Brasília, Distrito Federal, Brazil; b Department of Public Health, University of Copenhagen, Copenhagen, Denmark; c Department of Geography, University of Toronto Mississauga, Mississauga, Ontario, Canada; d Department of Environmental Health, Harvard TH Chan School of Public Health, Boston, MA, United States

**Keywords:** Wildfire, Smoke, Air pollution, Birth weight, Pregnancy

## Abstract

**Background:**

Air pollution exposure has been associated with critical neonatal morbidities, including low birth weight (LBW). However, little is known on short-term exposure to wildfire smoke and LBW. In this study, we estimated the association between birth weight following pregnancy and wildfire smoke exposure in more than 1.5 million newborns in Brazil (considered as a very fire-prone region worldwide).

**Methods:**

We applied a logistic regression model to estimate the percent variation in newborns with low birth weight when exposed to wildfire in different trimesters of the pregnancy.

**Findings:**

After adjusting the model with relevant covariates, we found that an increase of 100 wildfire records in Brazil was associated with an increase in low birth weight in the Midwest region [0.98% (95%CI:0.34; 1.63)] and in the South region [18.55% (95%CI:13.66; 23.65)] when the exposure occurred in the first trimester of pregnancy.

**Interpretation:**

Wildfires were associated with LBW and this should be of public health concern for policymakers.

**Funding:**

Brazilian Agencies National Council for Scientific and Technological Development (CNPq); Ministry of Science, Technology and Innovation in Brazil (MCTI); and Novo Nordisk Foundation Challenge Programme.

**Research in context**

**Evidence before this study**

To our knowledge, the first study on the relationship between air pollution and birth weight was performed by Alderman et al.^1^ Since then, especially in the last decade, this topic has been investigated in several studies across numerous regions, including California - USA,^2^ Sydney – Australia,^3^ Guangdong - China,^4^ and Rio de Janeiro - Brazil.^5^ Overall, most of the previous epidemiological investigations indicate that the evidence on the relationship between air pollution and birth weight suggests causality, but more studies were needed to assess the importance of exposure of different air pollution sources, including wildfire. In this context, existing studies on the effects of wildfire exposure on birth weight are relatively small. More importantly, these studies were mainly conducted in high-income countries and few in low and middle income countries like Brazil.

**Added value of this study**

Our findings add strength to the evidence that mother's exposure to air pollution during pregnancy may cause low birth weight. In our study, we found this evidence accounting for wildfire-related air pollution, which, to our knowledge, was not explored to date in South America. Also, our sample was relatively large, including more than 1.5 million records of birth nationwide over 18 years. To our best knowledge, this is the study with the largest sample size and the largest study period in Brazil.

**Implications of all the available evidence**

This study adds significantly to the growing evidence that wildfire-related air pollution might be harmful to fetus development and increase the risk of low birth weight. Given that wildfire is a growing problem in several regions worldwide, especially in Brazil – a fire-prone region, the epidemiological evidence shown in our study should be of great concern to the public health community and policymakers.

Alt-text: Unlabelled box

## Introduction

Environmental exposure has been associated with numerous adverse pregnancy outcomes, including low birth weight.^6^ Epidemiological observations have shown that infants born at low birthweight (according to the International Classification of Diseases 10th revision, defined as weight at birth of less than 2500 g) are more likely to experience a range of poor health outcomes which may increase the risk of death during the first year of life^7^ and even in the later childhood and adolescence.^8^ For those babies who survived during this period, they will have a greater risk of later adult chronic medical conditions, such as diabetes,^9^ hypertension,^10^ obesity,^9^ lower IQ,^11^ asthma,^10^ and heart disease.^12^

There are wide disparities in the prevalence of low birth weight among different population groups. About 20.5 million infants worldwide (approximately 15% of all births) were born in 2015 with low birth weight, of which 95% of them were born in developing countries.^13^ In Brazil, according to the World Health Organization (WHO), there were nearly 250 thousand infants born in 2015 with low birth weight, representing about 10% of all births in Brazil.^14^

Among the environmental factors related to low birth weight, a large body of literature has pointed out mother's exposure to air pollution during pregnancy as a significant public health concern. To our knowledge, the first study on the relationship between air pollution and birth weight was performed by Alderman et al.^1^ Since then, especially in the last decade, this topic has been investigated in several studies. In California - USA, ambient air pollution was associated with decreased birth weight – it was estimated a decrease of 12.8 g per 10 µg/m^3^ of PM_2.5_ and 5.7 g per ppm O_3_.^2^ In Sydney, Australia, an increase of 1 µg/m^3^ in mean PM_2.5_ was associated with a reduction of 4 g in birth weight.^3^ In Guangdong, China, exposure to PM_2.5_, NO_2_, and O_3_ in the first and last month of the pregnancy was associated with an increase in the risk of low birth weight.^4^ In the city of Rio de Janeiro, Brazil, exposure to O_3_ in the third semester of pregnancy was associated with a decrease of 17% in birth weight.^5^

Biological mechanisms involved in the effect of wildfire-related PM_2.5_ on birth weight are not well understood. The current body of evidence has focused on the toxicologic and physiologic effects of ambient air pollution on fetus development, without accounting for specific particulate matter sources – e.g., wildfire. The existing evidence shows that ambient air pollution may impact the anatomy and morphology of the placenta, causing reduced oxygen transport and increased blood viscosity,^15^ resulting in adverse fetal development,^16^ including fetal growth restriction.^17^ Besides the direct effect on the placenta, there is also evidence suggesting physiologic changes in pregnant women which creates vulnerability to their fetuses. Women during pregnancy have their alveolar ventilation rate increased by about 50%, resulting in an increased uptake of inhaled pollutants.^18^ With this increased inhalation, particles can enter the bloodstream of the pregnant women leading to oxidative stress, DNA damage, and as consequence, reduce fetal nutrient uptake.^19^ There is also evidence showing that the inhaled particles into the bloodstream of the pregnant women can cross the placenta and enter fetal circulation.^20^

Overall, most of the previous epidemiological investigations indicate that the evidence on the relationship between air pollution and birth weight suggests causality, but more studies are needed to assess the importance of exposure in different periods of pregnancy and the influence of different pollutants, including the variation by air pollution sources. Given that (i) ambient air pollution represents a heterogeneous mixture of chemical elements from different sources - e.g., fossil fuel combustion, industrial emissions, and biomass burning and (ii) health effects of air pollution is driven by the chemistry of air pollution toxicity which varies through air pollution sources over space and time, we focused on the exposure to wildfire smoke as an indicator of air pollution emission with a distinct characteristic in the chemistry of air pollution toxicity, and episodic occurrence. We estimated the association between birth weight following pregnancy and wildfire smoke exposure in more than 1.5 million infants in Brazil.

Further epidemiological studies on this topic are essential, given that wildfires have burned a large number of areas in the past years. Mouillot and Field^21^ estimate that about 6 million km^2^ of vegetation area is burned each year globally. Brazil is a very fire-prone region where, according to the National Institute of Spatial Research - INPE (http://queimadas.dgi.inpe.br/queimadas/), between January/2020 and August/2020 there were about 120,000 km^2^ of burned area. Wildfires emit substantial amounts of air pollutants that can travel over large distances, affecting air quality and human health far from the originating fires.^22^ Fine particulate matter (PM_2.5_) is the major pollutant emitted by wildfires. About 12–16% of global wildfire-related particulate emissions occur across Brazil.^23^

## Methods

### Birth data

Birth data were provided by the Ministry of Health in Brazil. This data includes individual records of birth in Brazil between 1 January 2001 and 31 December 2018. Birth data include event date, birth weight (grams, g), mother's home municipality, mother's age, mother's race (categorized as white, black, and indigenous), gestational age (categorized as the number of weeks of gestation), date of the last menstrual period, and sex of the infants. This data was obtained from publicly available database curated by the Ministry of Health in Brazil. The Brazilian Ministry of Health does not require ethical approval or informed consent for secondary analysis of anonymized health data at municipality level.

For this analysis, our inclusion criteria were: (1) infants born between the 37th and the 41st week of gestation; Given that preterm births (gestational age < 37 weeks) are correlated with low birth weight, this inclusion criteria will remove the effect of preterm birth on low birth weight; This inclusion criteria have been used by numerous studies.^2^^,^^24^, ^25^, ^26^, ^27^, ^28^ (2) birth records with birth weight between 1000 and 6000 g (as mentioned above, medical community defines birth weight as weight at birth of less than 2500 g); Infants born with less than 1000 g were removed from the analyses to exclude the outliers and minimize the bias; and (3) mothers aged > 18 years old, and mother aged < 45 years of age. There are studies suggesting adverse effects of young and advanced maternal age on neonatal outcomes, including low birth weight.^29^, ^30^, ^31^, ^32^, ^33^, ^34^, ^35^, ^36^ As a result of this subset, there were a total of 1,602,471 records of birth, of which 80,124 were cases and 1,522,347 were controls.

Regarding the mother's home location, the smallest spatial information included in the data is at the municipality level. There are 5572 municipalities in Brazil, which represent the smallest areas considered by the Brazilian political system. The government groups the municipalities by five regions, including the North, Northeast, Midwest, Southeast, and South. In Appendix 1 we show the spatial distribution of all municipalities and regions in Brazil. This Appendix gives a perspective about the size of the municipalities and regions in Brazil.

### Exposure data

Wildfire data were provided by the National Institute of Spatial Research of Brazil – *Instituto Nacional de Pesquisas Espaciais - INPE* (http://queimadas.dgi.inpe.br/queimadas/). The data include the date of wildfire records and its geographical location. These data are derived from seven satellite remote sensing observations, including NOAA-18, NOAA-19, METOP-B, MODIS (NASA TERRA and AQUA), VIIRS (NPP-Suomi and NOAA-20), GOES-16, and MSG-3. All images from these satellites are processed by INPE to estimate wildfire records. This estimate is based on a specific satellite as reference – AQUA satellite. Each wildfire record estimated here indicates the existence of fire within a pixel of 1 × 1 km. The minimum size of the wildfire to be detected by the satellite sensor is about 30 × 30 m. The quality of the data over time is high, given that the data is based on seven satellite, and then the estimates are derived from a reference satellite. We accounted for all wildfire records in Brazil based on the reference satellite in the period between 2000 and 2018. Then, we summed the daily wildfire records within each municipality in Brazil to merge the wildfire data with the health data. Note that the wildfire data is a continuous variable representing the number of wildfire records per day and per municipality.

### Covariates

We adjusted the analysis for ambient air pollution, meteorological variables, and socioeconomic variables included in the birth data (as mentioned above).

For ambient air pollution, we accounted for PM_2.5_, given the substantial amount of PM_2.5_ emitted by wildfires. At the global scale, approximately 32% of the total tropospheric organic aerosol in biomass burning aerosol originates from direct particulate emissions, whereas the rest is formed in the atmosphere.^37^ Specifically in Brazil, previous studies have reported validity for fire-generated PM_2.5_ in Brazil.^23^^,^^38^

Concentrations of PM_2.5_ (μg/m³) were obtained from a global model based on satellite observations. The data was accessed from the Copernicus Atmosphere Monitoring Service (CAMS), which include CAMS-Reanalysis predictions for the period between 2000 and 2018. The CAMS service runs ensemble models using several satellite observations and emission inventories amongst other predictors. The predictions were retrieved at a spatial resolution of 0.125° (approximately 12.5 km) and a temporal resolution of 6 h, including daily measurements for 00, 06, 12, and 18 UTC (Universal Time Coordinated). We calculated the daily mean temporal resolution from 2000 to 2018 and the daily mean concentration within each Brazilian municipality. Description of the validation of the CAMS global model is presented in Appendix 2.

For the meteorological data, it was accessed from the European Centre for Medium-Range Weather Forecasts (ECMWF). Weather data include surface temperature (°C), humidity (%), wind speed (m/s), wind direction (°), and precipitation (mm/day). Temperature, humidity, wind speed, and wind direction were derived from Era-Interim reanalyses, with a spatial resolution of 0.125° and temporal resolution of 6 h. This reanalysis was performed by the ECMWF. Precipitation data was accessed from the Climate Prediction Center (CPC) and the National Ocean and Atmospheric Administration (NOAA). This data has an original spatial resolution of 0.50° (approximately 50 km), with interpolation to 12.5 km, and a temporal resolution of 6 h. We accounted for daily mean values of weather variables within each Brazilian municipality.

### Exposure assignment

The wildfire exposure window was based on the gestational intervals, which included the first (week 1 to week 12), the second (week 13 to week 28), and the third trimesters (week 29 to week 37). For each trimester, we estimated the sum of wildfire records – the exposure variable. This estimate was based on the number of wildfire occurrences within the boundaries of the mother's home municipality. We also calculated the average PM_2.5_ and meteorological covariates for each trimester within the boundaries of the mother´s municipalities.

Note that in our study design we considered birth weight because of a gradual crossover, meaning that it is plausible that several weeks of exposure are needed to manifest the effect. We highlight that the use of trimester exposure can be used to compare the weekly averages with the trimester averages. If they are similar, we can assume that there are no bias and trimester exposures are more clinically relevant.

### Statistical analyses

We applied a case-control study design using logistic regression model to estimate the odds ratio (OR) for low birth weight associated with wildfire occurrence during a specific trimester of pregnancy (1–3 trimester). The cases were defined as newborns with weights between 1000 and 2500 g and controls were newborns with weights more than 2500 g to 6000 g.

We adjusted the model for the following confounding variables: PM_2.5_, meteorological variables (precipitation, temperature, relative humidity, wind direction, and wind speed), mother's age, mother´s home state (as a spatial term), year of birth (as a temporal term), and latitude/longitude (centroids) of the mother's home municipality. We adjusted de model for PM_2.5_ because of the biological pathway. There may be at least two types of etiologic pathways possibly linking prenatal wildfire exposure with low birth weight – biological pathway (exposure to air pollution from wildfires) and psychosocial pathway (stress caused by direct or indirect consequences of the wildfires). It is also possible a mixture of these two pathways. The Centers for Disease Control and Prevention (CDC) has recognized these aspects and released guidelines (https://www.cdc.gov/air/wildfire-smoke/pregnancy.htm). Therefore, we used the wildfire records as the main exposure because we cannot measure the ecological indicator of maternal stress (psychosocial pathway). Controlling for PM2.5 we can capture the effects from exposure to air pollution from the fires (biological pathway). Consequently, our results may reflect the potential conjoint impact of these two pathways. The mode was given by:

$$\begin{array}{c} logit\left(LBW_{j,\;t}\right)=\alpha +\beta_{1}\;wildfire_{t}+\;s_{1}{\left(PM_{2.5}\right)}_{t}+\;s_{2}{\left(temperature\right)}_{t}+s_{3}{\left(humidity\right)}_{t}+s_{4}{\left(precipitation\right)}_{t}\\ +s_{5}{\left(Wind\;Speed\right)}_{t}+s_{6}{\left(Wind\;Direction\right)}_{t}+\;s_{7}\;age+s_{8}\;year+\delta \;\text{state}\\ +s_{9}\left(latitude,longitude\right)+\;e_{j,\;t} \end{array}$$

where, *LBW* is the probability of low birth weight in the region *j*, during the trimester of exposure *t*; α is the regression intercept; *β* values are regression coefficients for the exposure variable wildfire (during the trimester *t*); *s()* are the smoothing spline function to characterize nonlinear relationships between LBW and the confounders PM_2.5_, weather parameters, age (mother's age), year (year of birth), and latitude/longitude (centroids of the mother's home municipality) during the trimester *t*; δ is the vector of coefficient that represents the variability by state; and *e* is the error term spatially and temporally dependent based on the regions *j* and trimester of exposure *t.* We used R - version 2.13.1 (R Core Team, 2013) to perform the statistical analysis. We performed the logistic regression models using the GLM function. To allow the inclusion of spline function in the model, we used the package “splines”. We report the results as percentage change in risk (and 95% CI) of low birth weight associated with an increase of 100 wildfire records. The percentage change in risk was calculated as (OR−1) × 100%. We used this approach to describe the health burden associated with exposure, which may be a better information for policy makers and general readers (Steenland and Armstrong 2006). We conducted a sensitivity analysis by stratifying the analyses by sex and by race (white and black). We conducted the analyses for each one of the five Brazilian regions (Appendix 1 shows the spatial distribution of these regions). We performed this subgroup analysis by region to capture the regional heterogeneity of landscape in Brazil (e.g., Amazon Forest, Atlantic Forest, Pantanal, etc.), which is strongly correlated with wildfire occurrences.

*Role of the funding source:* The funding bodies did not play any role in the study design, data collection, data analyses, results interpretation, writing of this manuscript or decision to publish.

## Results

### Characteristics of the birth and exposure data

Table 1 shows the descriptive statistics for the study population which is composed of 1,602,471 observations. Overall, majority of the infants in our study population were girls and white over the five Brazilian regions. Southeast was the region with the highest number of infants (663,408 infants, representing 41% in Brazil). Midwest was the region with the fewest number of infants, including a total of 120,343 newborns, representing 7.5% of the whole dataset.

**Table 1:** Descriptive characteristics of the birth data in Brazil, 2001–2018.

Region	Subgroup	*n* (%)^1^
North	Infant sex: Male	70,634 (43.2)
	Infant sex: Female	92,752 (56.7)
	Infant sex: NA	131 (0.1)
	Maternal race: white	152,535 (93.3)
	Maternal race: black	2,502 (1.5)
	Maternal race: indigenous	6,748 (4.1)
	Maternal race: NA	1,732 (1.1)
		163,517
Northeast	Infant sex: Male	195,531 (42.7)
	Infant sex: Female	261,784 (57.2)
	Infant sex: NA	577 (0.1)
	Maternal race: white	400,308 (87.4)
	Maternal race: black	14,584 (3.2)
	Maternal race: indigenous	1,611 (0.4)
	Maternal race: NA	41,389 (9)
		457,892
Midwest	Infant sex: Male	50,057 (41.6)
	Infant sex: Female	70,196 (58.3)
	Infant sex: NA	90 (0.1)
	Maternal race: white	101,407 (84.3)
	Maternal race: black	2,712 (2.3)
	Maternal race: indigenous	1,865 (1.5)
	Maternal race: NA	14,359 (11.9)
		120,343
Southeast	Infant sex: Male	274,846 (41.4)
	Infant sex: Female	388,096 (58.5)
	Infant sex: NA	466 (0.1)
	Maternal race: white	574,428 (86.6)
	Maternal race: black	31,285 (4.7)
	Maternal race: indigenous	782 (0.1)
	Maternal race: NA	56,913 (8.6)
		663,408
South	Infant sex: Male	81,072 (41.1)
	Infant sex: Female	116,178 (58.9)
	Infant sex: NA	61 (0)
	Maternal race: white	188,368 (95.5)
	Maternal race: black	7,186 (3.6)
	Maternal race: indigenous	610 (0.3)
	Maternal race: NA	1,147 (0.6)
		197,311

Notes: (1) percentage was based on the proportion of observations by region.

Note: the categories with “NA” are for those observations with missing value for the categorial variables representing sex and race.

**Figure 1:**
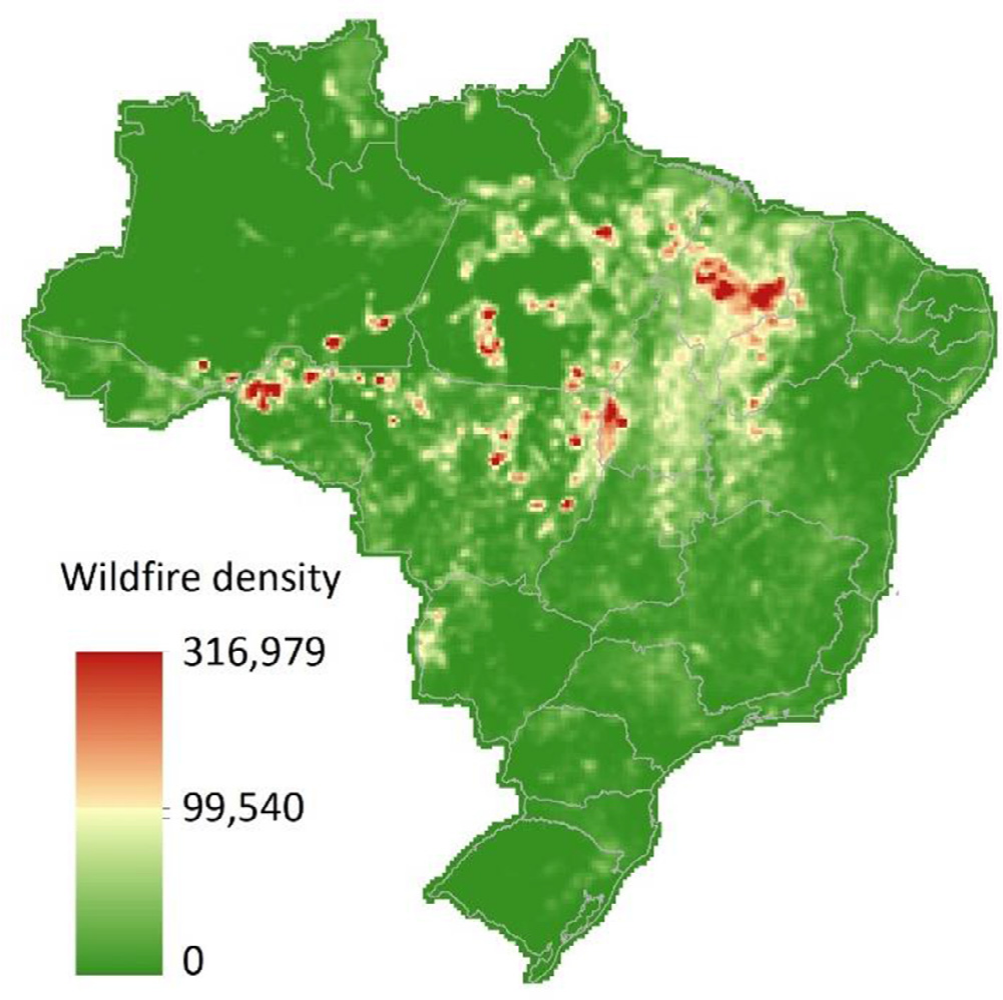
Nationwide concentration of wildfire density (based on Kernel density with an output cell size of 0.15°; here we accounted for all wildfire records over the study period within a radius of 0.28°) in Brazil.

Summary statistics for wildfire and covariates stratified by regions and trimesters are shown in Table 2. North is the region with the highest wildfire records, with a maximum value exceeding 12 thousand wildfire records and an average value varying between 23 and 29 wildfire occurrences over the trimesters. The highest concentration of ambient PM_2.5_ is also in the North region, with an average value between 37 and 39 μg/m³ over the trimesters. Overall, the summary statistics for wildfire and covariates throughout the trimesters are similar. In Figure 1 we illustrate the nationwide spatial distribution of wildfire density.

**Table 2:** Summary statistics for wildfire, air pollution, and weather by region, 2001–2018.

		First trimester				Second trimester				Third trimester			
Region	Variable	Min.	Mean	SD	Max.	Min.	Mean	SD	Max.	Min.	Mean	SD	Max.
North	Wildfire records	0	23.72	161.95	9,114	0	24.74	176.87	12,813	0	29.49	216.01	12,822
	PM_2.5_ (μg/m³)	2.02	39.01	39.15	461.86	2.03	37.65	38.89	472.20	1.91	37.70	40.01	485.00
	Temperature (°C)	22.35	26.28	0.98	29.95	22.33	26.31	0.97	29.95	22.34	26.36	1.02	29.95
	Relative humidity (%)	38.00	84.80	8.01	95.49	37.96	84.73	7.96	95.49	37.84	84.32	8.37	95.49
	Wind speed (m/s)	0.98	1.99	0.54	6.60	0.98	1.99	0.55	6.59	0.98	2.01	0.56	6.60
	Wind direction (°)	35.79	108.32	42.72	288.55	36.15	108.20	42.71	287.71	35.70	108.01	42.53	288.51
	Preciptation (mm/day)	0.00	5.91	3.64	29.26	0.00	5.89	3.66	29.34	0.00	5.81	3.71	29.34
Northeast	Wildfire records	1	25.13	65.02	1,057	1	24.85	66.33	1,109	1	25.46	65.13	1,003
	PM_2.5_ (μg/m³)	2.02	18.51	23.50	217.43	2.10	18.77	23.54	229.87	2.06	18.71	23.49	213.52
	Temperature (°C)	19.20	25.95	1.84	31.76	19.33	26.03	1.82	31.77	19.27	26.02	1.84	31.87
	Relative humidity (%)	38.56	74.52	9.75	93.82	38.48	74.41	9.77	94.44	38.14	74.42	9.87	93.87
	Wind speed (m/s)	1.36	3.88	1.13	8.60	1.37	3.86	1.11	8.58	1.34	3.85	1.11	8.58
	Wind direction (°)	43.74	109.84	20.82	200.24	43.74	108.94	20.63	204.25	43.33	109.34	20.72	200.05
	Preciptation (mm/day)	0.00	3.22	2.85	18.21	0.00	3.22	2.78	18.73	0.00	3.27	2.79	18.73
Midwest	Wildfire records	0	14	100	4,116	0	14	105	5,622	0	14	115	5,642
	PM_2.5_ (μg/m³)	2.25	32.52	40.96	395.69	2.21	30.58	39.90	395.79	2.21	28.59	37.35	395.23
	Temperature (°C)	16.82	24.18	1.84	30.30	17.19	24.26	1.80	30.30	17.07	24.25	1.81	30.30
	Relative humidity (%)	37.73	67.06	10.86	95.39	37.85	67.46	10.80	95.39	37.74	67.35	11.06	95.39
	Wind speed (m/s)	1.13	2.53	0.41	4.39	1.13	2.52	0.41	4.39	1.13	2.52	0.41	4.33
	Wind direction (°)	88.38	142.62	28.63	291.61	88.18	143.55	29.12	291.28	88.38	143.40	29.12	289.44
	Preciptation (mm/day)	0.00	3.87	2.96	17.44	0.00	4.01	2.96	17.50	0.00	3.96	3.00	17.54
Southeast	Wildfire records	1	7	14	285	1	7	13	289	1	7	14	324
	PM_2.5_ (μg/m³)	2.64	26.45	19.38	120.58	2.81	26.70	20.66	119.24	2.64	26.82	21.33	121.48
	Temperature (°C)	14.47	21.72	2.38	27.92	14.85	21.95	2.31	27.76	14.58	21.95	2.33	27.87
	Relative humidity (%)	43.87	76.60	6.40	88.19	44.88	76.96	6.27	88.34	45.19	77.13	6.20	88.53
	Wind speed (m/s)	1.47	2.72	0.51	6.29	1.56	2.71	0.51	6.23	1.53	2.70	0.50	6.17
	Wind direction (°)	65.49	144.82	26.11	219.28	66.09	144.92	26.12	218.72	65.02	145.31	26.06	219.82
	Preciptation (mm/day)	0.00	3.72	2.46	16.74	0.00	3.95	2.47	13.90	0.00	3.89	2.49	16.10
South	Wildfire records	0	1	9	560	0	1	9	591	0	1	9	585
	PM_2.5_ (μg/m³)	4.03	25.67	20.53	171.03	4.07	23.69	19.69	174.59	4.04	21.45	17.11	171.03
	Temperature (°C)	10.80	19.47	3.30	27.32	11.33	19.71	3.17	27.24	11.33	19.66	3.19	27.29
	Relative humidity (%)	56.22	79.27	4.63	88.93	55.98	79.41	4.65	88.92	56.29	79.57	4.51	88.94
	Wind speed (m/s)	1.54	3.09	0.74	8.43	1.56	3.07	0.74	8.43	1.54	3.08	0.74	8.37
	Wind direction (°)	92.83	150.21	20.51	245.03	92.78	149.28	20.34	238.95	92.78	149.53	20.61	238.52
	Preciptation (mm/day)	0.10	4.43	1.63	16.20	0.14	4.46	1.64	16.20	0.14	4.41	1.63	16.09

Note: minimum (Min.), Standard Deviation (SD), maximum (Max.).

### Percentage variation in infants with low birth weight when exposed to wildfire

**Figure 2:**
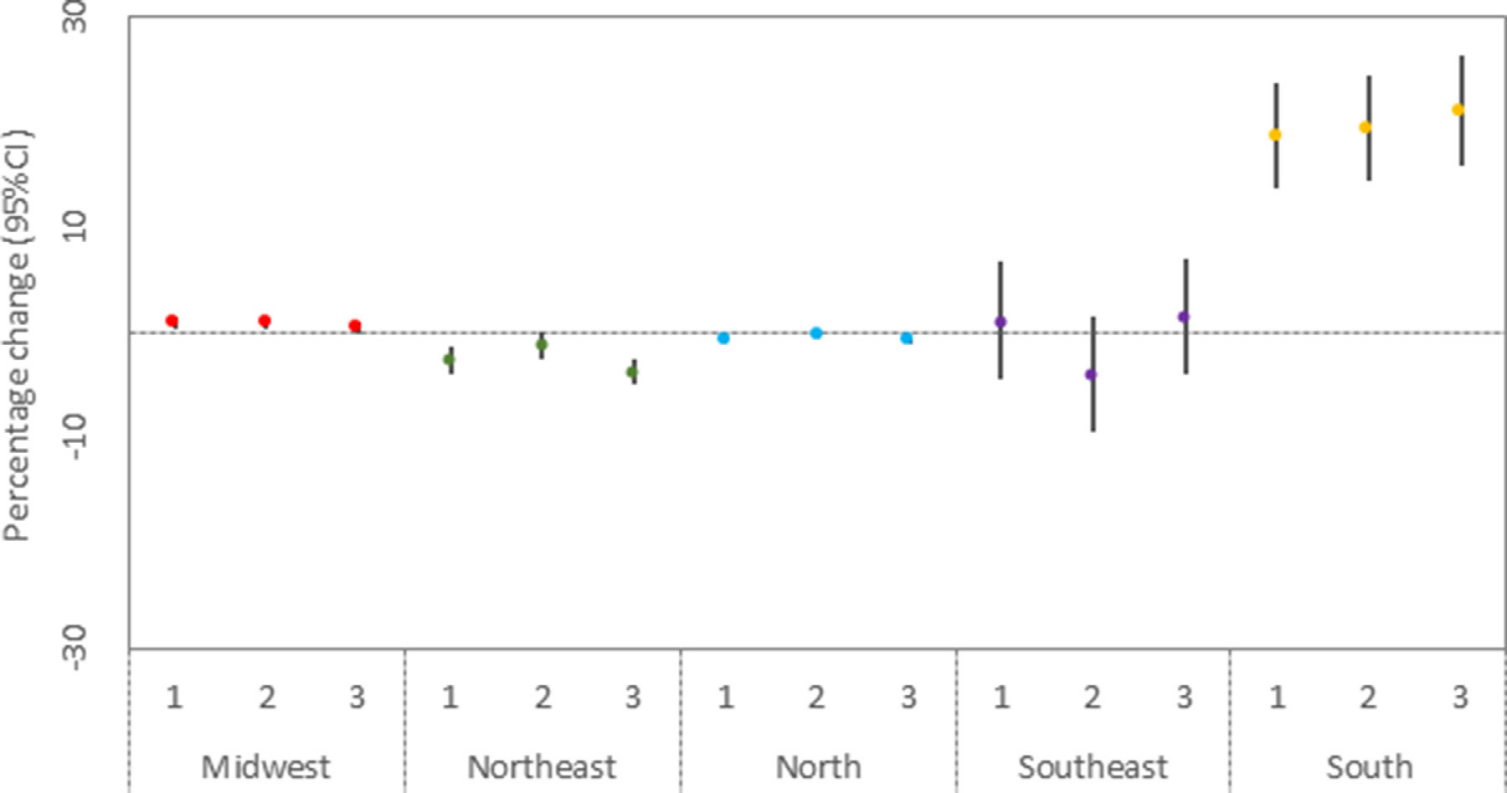
Regional percentage increase in ODDs (and 95% CI) of low birth weight associated with an increase of 100 wildfire records for trimesters 1, 2, and 3. Note: numbers in the *x*-axis indicate the trimesters.

Figure 2 shows the results of the primary analysis stratified by region and trimesters. South was the region with the highest risk of low birth weight associated with wildfire, with an estimated increase of 18.55% (95%CI: 13,66–23,65%) in low birth weight when the exposure occurred in the first trimester. Our results showed a variation of the associations depending on the trimester of exposure. Overall, the highest associations occurred in the first and third trimesters.

**Figure 3:**
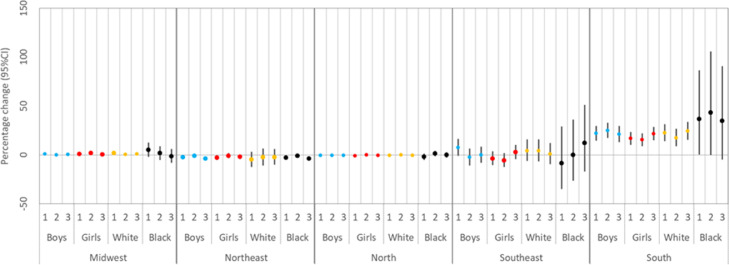
Regional percentage increase in ODDs (and 95% CI) of low birth weight associated with an increase of 100 wildfire records for the trimesters 1, 2, and 3, stratified by sex and race. Note: *x*-axis indicates the trimesters.

The subgroup analysis by sex and race (only white and black) are shown in Figure 3. The results from the subgroup analysis by sex and race show a substantial heterogeneity of the risk of low birth weight associated with wildfire exposure across regions.

## Discussion

Estimating the health effects of different particulate matter sources and constituents is suggested as a priority by the environmental health community. For example, this is one of the priorities listed by the Health Effects Institute (HEI) in the strategic plan for understanding the health effects of air pollution (https://www.healtheffects.org/about/strategic-plan). Our study is in line with this essential concern by contributing to the scientific evidence on health effects on fetus development (birth weight) due to exposure to a specific particulate source (wildfire smoke). This contribution is essential because, on an equal-mass basis, wildfire-related PM_2.5_ may be more toxic than ambient PM_2.5_ in the same region during non-fire periods,^39^ due to the formation of secondary pollutants as a result of the atmospheric photochemistry.^40^ Our findings suggest that wildfire exposure in Brazil is associated with an increase in low birth weight. This is consistent with the sparse literature on the effects of wildfire exposure on birth outcomes. Bell et al.^25^ focused on the association between birth weight and numerous PM_2.5_ elemental constituents in Massachusetts and Connecticut in the US. They found that an interquartile range increase in exposure was associated with low birth weight for various PM_2.5_ chemical constituents, including elemental carbon (13% increase in risk). Particulate carbon (including the organic and elemental carbons) has been the most indicated as trace elements of wildfires.^41^ In the state of Colorado in the US, between the years 2007 and 2013, the wildfires were associated with 3.4% increases in low birth weight.^44^ In Brazil, to our knowledge, there is only one study on this topic,^27^ which accessed the impacts of particulate matter and carbon monoxide (here the authors used this pollutant as an indication of wildfire emissions) exposure on birth weight accounting for 6,147 birth records (we accounted for 1,602,471 records) in cities within the state of Mato Grosso in the Midwest region (we accounted for the whole Brazil). The authors estimated an OR of 1.49 (95CI: 1.03–2.14) associated with the 4th quartile of CO.

Our findings showed that different trimesters of wildfire exposure during pregnancy may play different roles in fetal growth. We found that the exposures during the first and the third trimester were associated with the highest associations. Previous studies estimated mix results, including the Brazilian study mentioned above,^27^ in which the third trimester was identified as the most critical; a study in California,^28^ which the second and the third trimester were the most critical; another study in California,^43^ which the third trimester was the most important; a study in China,^44^ which the third trimester was the most critical; and an investigation in Colorado, which only the exposure during the first trimester presented significant associations.^45^ A recent review study suggests that the exposure in the late pregnancy suggest important evidence on birth weight reduction.^46^ First-trimester growth and the risk of low birth weight is supported by the theoretical framework regarding the biological mechanisms, the weeks 4 through 8 represent the embryonic period, when occurs the formation of all major organ systems.^47^ Therefore, the biological mechanism does not support the associations found in our study when the exposure occurred in the third trimester. To check this issue, we looked at the OR of third trimester adjusting for first trimester exposure, and we observed that the effect disappeared.

We also observed distinct impact of exposure among racial groups. This is more evident for the black Southern group, suggesting an acute effect comparing with the white group (Figure 3). It would be very speculative if we propose an explanation for this acute finding for the black Southern group without a deep analysis of the socio-economic/demographic condition in Brazil. Further studies should explore this issue.

Our findings should be interpreted considering some limitations. First, the location of the wildfire may have resulted in some exposure measurement error. This may create bias in the OR estimates and affect the accuracy of our results. Second, there is a possibility of some residual confounding error, even after the adjustment for multiple spatiotemporal factors and status variables in our model. There is still a lack of important individual information, including smoking, obesity, etc. The potential implication of this limitation is related to the inaccurate estimate (underestimate or overestimate) of the true association between wildfire exposure and birth weight. Third, our results only suggest an association between exposure to wildfire and low birth weight – this approach does not capture the cause-effect between wildfire exposure and birth weight.

Our study, however, has several strengths. First, our findings add strength to the evidence that mother's exposure to air pollution during pregnancy may cause low birth weight. In our study, we found this evidence accounting for wildfire-related air pollution, which, to our knowledge, was not explored to date in South America. Second, our sample was relatively large, including more than 1.5 million records of birth nationwide for over 18 years. This is the study with the largest sample size and the largest study period in Brazil. Due to this large sample, our statistical analysis had enough power to be able to detect differences in outcome measures. Third, the birth dataset included many individual-specific variables for the assessment of potential confounding.

In conclusion, this study demonstrates that wildfire exposure may increase the risk of infants with low birth weight. Given that wildfire is a growing problem in several regions worldwide, especially in Brazil – a fire-prone region, the epidemiological evidence shown in our study should be of great concern to the public health community and policymakers.

## Contributors

WJR contributed to the conceptualization, methodology, data curation, funding acquisition, and prepared the original draft. HA contributed towards the exposure assessment, health analysis, and review and editing of the manuscript. MDA participated in reviewing and editing of the manuscript and contributed towards the exposure analysis. JDS contributed towards the methodology and reviewing and editing of the manuscript.

## Data sharing statement

Researchers who are interested in using the data should contact Dr. Requia (weeberb.requia@fgv.br). There will be an assessment for the data request by a committee including stakeholders from the Brazilian Ministry of Health. If approved by the committee, the researcher can gain access to the data.

## Editor note

The Lancet Group takes a neutral position with respect to territorial claims in published maps and institutional affiliations.

## Declaration of interests

None.

## Appendix Supplementary materials

Image, application 1
