# Supplementary material for: Birth weight following pregnancy wildfire smoke exposure in more than 1.5 million newborns in Brazil: A nationwide case-control study
Source: Lancet Reg Health Am. 2022 Mar 15;11:100229. doi: 10.1016/j.lana.2022.100229 (PMC9903686; doi:10.1016/j.lana.2022.100229)
Supplement: Supplementary file 1 [file mmc1.docx]

**SUPPLEMENTARY MATERIALS**

**Birth weight following pregnancy wildfire smoke exposure in more than 1.5 million babies in Brazil: A nationwide case-control study**

**Weeberb J. Requia**

(Corresponding Author)

School of Public Policy and Government, Fundação Getúlio Vargas

Brasília, Distrito Federal, Brazil

**Heresh Amini**

Department of Public Health, University of Copenhagen

Copenhagen, Denmark

**Matthew D. Adams**

Department of Geography, University of Toronto Mississauga

Mississauga, Ontario, Canada

**Joel D. Schwartz**

Department of Environmental Health, Harvard TH Chan School of Public Health

Boston, Massachusetts, United States

**Appendix 1**

Figure 1 (Appendix 1): Spatial distribution of the municipalities and regions in Brazil.

**
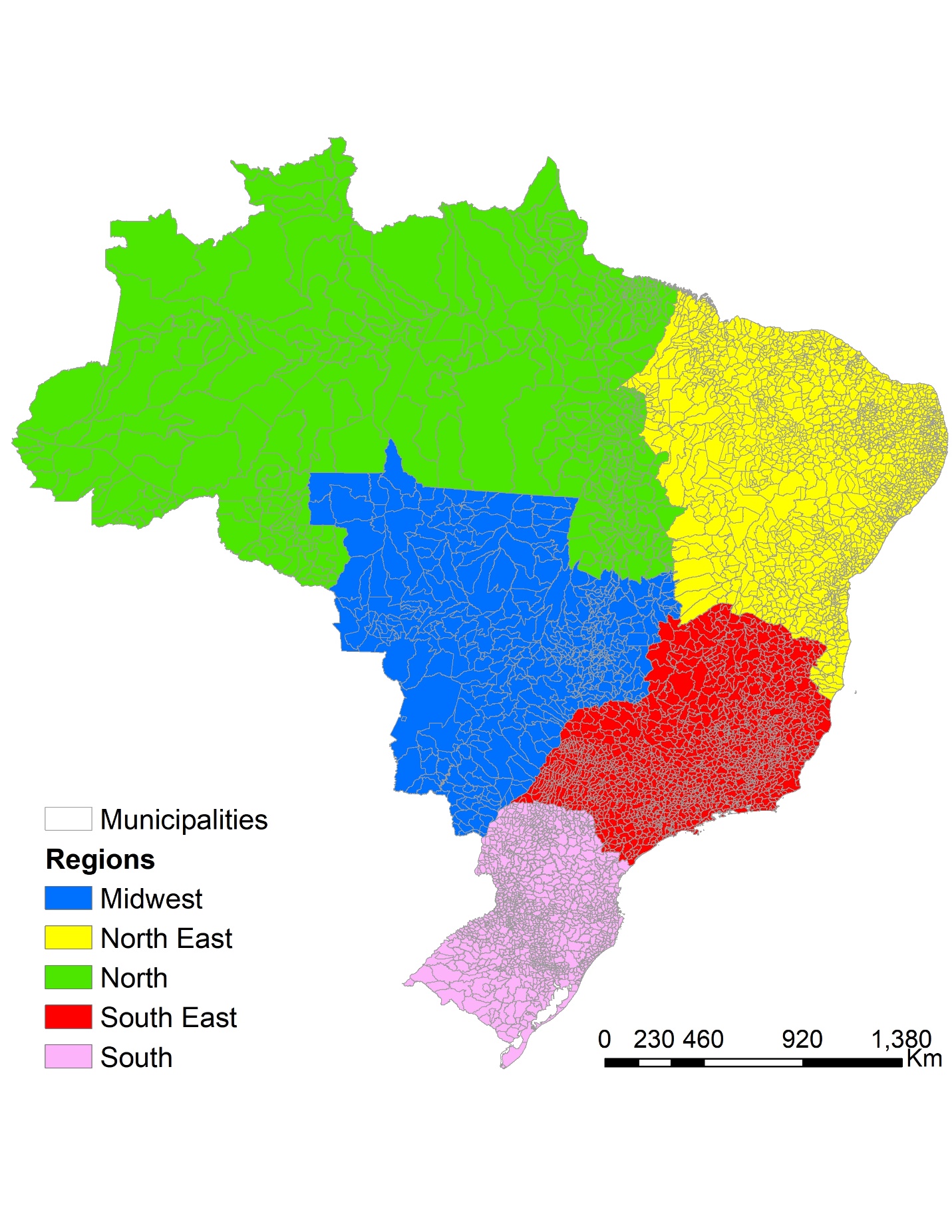
**

**Appendix 2 – Validation of the CAMS model**

The validation for the CAMS global model is reported by Inness et al. (2018). Specifically, for the PM_2.5_, the exposure variable in our study, it is evaluated with ground observations of the Aerosol Robotic Network (AERONET). There are over 500 AERONET stations worldwide measuring spectral Aerosol Optical Depth (AOD) with ground-based sun photometers. Among those AERONET stations, about 27 stations are in Brazil. The validation by Inness et al. (2018) estimated a mean bias and standard deviations from the data provided by the satellite’s instruments (included in the CAMS model for aerosols) relative to AERONET data. In South America, the data from satellite’s instruments are slightly smaller, with an approximate bias of -0.006 ± 0.128. Another investigation shows that CAMs estimates in South America have a root mean square error – RMSE (compared with AERONET stations) of 0.268 (Gueymard and Yang, 2020). Other studies have shown that AERONET observation sites in South America has significant representativity for AOD measured by Moderate Resolution Imaging Spectroradiometer (MODIS), aboard TERRA and AQUA satellites (Hoelzemann et al., 2009). Note that MODIS is an instrument included in the CAMS model. This association between AERONET data and AOD from MODIS is significant during the biomass burning seasons in South America, which the R^2^ (coefficient of determination) for most of the AERONET stations in Brazil was higher than 0.85 (Hoelzemann et al., 2009).

The relationship between AOD and PM_2.5_ is based on the remote sensing process of detecting aerosols (fine solid and/or liquid particles suspended in the air). In short, the radiation interacts with aerosols in the atmosphere resulting in distortion. This distortion is estimated by the radiative transfer model and can be converted into aerosol loading, defined as AOD (Kumar et al., 2007). Given that AOD and PM_2.5_ are based on the primary source (presence of aerosols in the atmosphere), there is a strong positive relationship between AOD and PM_2.5_. Several studies have explored this relationship (Naresh, 2010; Xie et al., 2015; Xu and Zhang, 2020; Yang et al., 2019). Models predicting fine particle using AOD retrievals and ground-based measurements have reported good performance across regions worldwide, including a cross-validated R^2^ of 0.73 in Switzerland (de Hoogh et al., 2018), 0.87 in China (Hu et al., 2019), 0.88 in Northeastern USA (Kloog et al., 2014), and 0.82 in Brazil (Gonçalves et al., 2018). The CAMS global model is considered one of the strongest models used in air pollution and epidemiological studies.

**References:**

de Hoogh, K., Héritier, H., Stafoggia, M., Künzli, N., Kloog, I., 2018. Modelling daily PM2.5 concentrations at high spatio-temporal resolution across Switzerland. Environ. Pollut. 233, 1147–1154. https://doi.org/https://doi.org/10.1016/j.envpol.2017.10.025

Gonçalves, K. dos S., Winkler, M.S., Benchimol-Barbosa, P.R., de Hoogh, K., Artaxo, P.E., de Souza Hacon, S., Schindler, C., Künzli, N., 2018. Development of non-linear models predicting daily fine particle concentrations using aerosol optical depth retrievals and ground-based measurements at a municipality in the Brazilian Amazon region. Atmos. Environ. 184, 156–165. https://doi.org/10.1016/j.atmosenv.2018.03.057

Gueymard, C.A., Yang, D., 2020. Worldwide validation of CAMS and MERRA-2 reanalysis aerosol optical depth products using 15 years of AERONET observations. Atmos. Environ. 225, 117216. https://doi.org/10.1016/j.atmosenv.2019.117216

Hoelzemann, J.J., Longo, K.M., Fonseca, R.M., Do Rosário, N.M.E., Eibern, H., Freitas, S.R., Pires, C., 2009. Regional representative of AERONET observation sites during the biomass burning season in South America determined by correlation studies with MODIS Aerosol Optical Depth. J. Geophys. Res. Atmos. 114, 1–20. https://doi.org/10.1029/2008JD010369

Hu, H., Hu, Z., Zhong, K., Xu, J., Zhang, F., Zhao, Y., Wu, P., 2019. Satellite-based high-resolution mapping of ground-level PM2.5 concentrations over East China using a spatiotemporal regression kriging model. Sci. Total Environ. 672, 479–490. https://doi.org/https://doi.org/10.1016/j.scitotenv.2019.03.480

Inness, A., Ades, M., Agusti-Panareda, A., Barré, J., Benedictow, A., Blechschmidt, A.-M., Dominguez, J.J., Engelen, R., Eskes, H., Flemming, J., Huijnen, V., Jones, L., Kipling, Z., Massart, S., Parrington, M., Peuch, V.-H., Razinger, M., Remy, S., Schulz, M., Suttie, M., 2018. The CAMS reanalysis of atmospheric composition. Atmos. Chem. Phys. Discuss. 1–55. https://doi.org/10.5194/acp-2018-1078

Kloog, I., Chudnovsky, A.A., Just, A.C., Nordio, F., Koutrakis, P., Coull, B.A., Lyapustin, A., Wang, Y., Schwartz, J., 2014. A new hybrid spatio-temporal model for estimating daily multi-year PM2.5 concentrations across northeastern USA using high resolution aerosol optical depth data. Atmos. Environ. 95, 581–590. https://doi.org/https://doi.org/10.1016/j.atmosenv.2014.07.014

Kumar, N., Chu, A., Foster, A., 2007. An empirical relationship between PM2.5 and aerosol optical depth in Delhi Metropolitan. Atmos. Environ. 41, 4492–4503. https://doi.org/https://doi.org/10.1016/j.atmosenv.2007.01.046

Naresh, K., 2010. What Can Affect AOD–PM2.5 Association? Environ. Health Perspect. 118, A109–A110. https://doi.org/10.1289/ehp.0901732

Xie, Y., Wang, Y., Zhang, K., Dong, W., Lv, B., Bai, Y., 2015. Daily Estimation of Ground-Level PM2.5 Concentrations over Beijing Using 3 km Resolution MODIS AOD. Environ. Sci. Technol. 49, 12280–12288. https://doi.org/10.1021/acs.est.5b01413

Xu, X., Zhang, C., 2020. Estimation of ground-level PM2.5 concentration using MODIS AOD and corrected regression model over Beijing, China. PLoS One 15, e0240430.

Yang, Q., Yuan, Q., Yue, L., Li, T., Shen, H., Zhang, L., 2019. The relationships between PM2.5 and aerosol optical depth (AOD) in mainland China: About and behind the spatio-temporal variations. Environ. Pollut. 248, 526–535. https://doi.org/10.1016/j.envpol.2019.02.071
